# Online mindfulness-based cognitive therapy for treatment-resistant depression: a parallel-arm randomized controlled feasibility trial

**DOI:** 10.3389/fpsyg.2024.1412483

**Published:** 2024-07-02

**Authors:** Michele Ferreira Rodrigues, Laiana Quagliato, Jose Carlos Appolinario, Antonio E. Nardi

**Affiliations:** Institute of Psychiatry (IPUB) of Federal University of Rio de Janeiro (UFRJ), Rio de Janeiro, Brazil

**Keywords:** treatment-resistant depression, mindfulness-based cognitive therapy, online therapy, digital mental health intervention, mindfulness and meditation

## Abstract

**Introduction:**

Treatment-resistant depression (TRD) presents a significant challenge, affecting approximately 30% of individuals diagnosed with major depressive disorder and leading to poor treatment responses. Innovations in digital mental health, especially online mindfulness-based cognitive therapy (eMBCT), offer promising avenues for enhancing access to effective mental health care for individuals with TRD in a clinical setting.

**Objective:**

The aim of this study was to examine the feasibility of eMBCT in an individual clinical context to decrease depressive symptoms for TRD.

**Methods:**

Conducted at the Institute of Psychiatry of the Federal University of Rio de Janeiro, Brazil, this parallel-arm, randomized controlled feasibility trial involved outpatients diagnosed with TRD, aged 18 and above. Of the 39 outpatients invited, 28 were randomized into two groups: an intervention group receiving the eMBCT program (*n* = 15) and a control group (*n* = 13). The intervention, consisting of an 8-week course, was delivered via live video sessions. Following the assessment period, participants in the control group were offered the eMBCT intervention. Assessments using standardized questionnaires were conducted at the start and end of the study.

**Results:**

Within the eMBCT group, improvements were observed in depression symptoms (*Z* = −3.423; *p* = 0.001; effect size *r* = 0.78), anxiety symptoms (*Z* = −3.361; *p* = 0.001; effect size *r* = 0.77), with no significant changes in the control group. Comparatively, the eMBCT group showed significant reductions in depression symptoms and improvements in clinical global impressions over the control group (BDI2: *U* = 30.5; *p* = 0.015; effect size *r* = 0.47, CGI1: *U* = 21.0; *p* = 0.004; effect size *r* = 0.56).

**Conclusion:**

eMBCT in an individual format combined with medication, appears to be a feasible treatment for TRD, decreasing symptoms of depression. In a future trial the control group may have a manualized intervention.

**Clinical trial registration:**

The Brazilian Clinical Trials Registry: (https://ensaiosclinicos.gov.br/rg/RBR-6zndpbv) and RBR-6zndpbv.

## 1 Introduction

Major depressive disorder (MDD) is a debilitating psychiatric condition associated with risk of morbidity and suicide (Cuijpers et al., 2014; Lundberg et al., 2023). Treatment-resistant depression (TRD) includes, in general, MDD patients who failed to fully remit from depressive symptoms after at least two antidepressant trials (Salloum and Papakostas, 2019). The demand for therapies treating TRD has increased, and mindfulness-based cognitive therapy (MBCT) is a promising alternative for treatment of mental disorders. Its prevalence has grown over the years as more individuals are suffering from the symptoms (McIntyre et al., 2023). Considering that chronically depressed patients have a lower chance of recovery, developing efficient treatment is a current necessity (Al-harbi, 2012; Voineskos et al., 2020). TRD may represent up to 30% of patients with major depression (Gaynes et al., 2011).

MBCT integrates mindfulness with cognitive behavioral therapy (CBT) with the aim of introducing a different way to connect the states of mind, causing a detachment from the automatic patterns of mind and body, therefore developing thoughts, feelings, and more consciousness (Segal et al., 2013). Originally conceptualized for the prevention of depressive relapse, the scope of MBCT has expanded to encompass the treatment of acute depression, recurrent episodes, and notably, TRD showing good outcomes (Eisendrath et al., 2011; Cladder-Micus et al., 2018; Monnart et al., 2019).

Traditional MBCT has been compared with various other treatment methods, such as pharmacotherapy and other forms of psychotherapy. Pharmacotherapy, often the first line of treatment for MDD, includes the use of antidepressants like SSRIs and SNRIs. While effective for many, a significant portion of patients do not achieve full remission, leading to TRD (Rush et al., 2006). Cognitive Behavioral Therapy (CBT) and Interpersonal Therapy (IPT) are other psychotherapeutic approaches that have shown efficacy in treating depression (Cuijpers et al., 2013). However, MBCT offers unique advantages by combining mindfulness practices with cognitive strategies, which can help patients develop a different relationship with their thoughts and emotions, potentially reducing the risk of relapse (Kuyken et al., 2016).

The integration of the Internet into daily life has catalyzed a shift toward digital mental health interventions, with a notable rise in the preference for treatments delivered online (Ramos et al., 2024). Online MBCT in an individual format is following this trend of preference when compared to treatment delivered face-to-face. A survey involving 500 adults showed a significant preference for individual (*n* = 384) and online (*n* = 356) mindfulness meditation interventions over group settings (*n* = 245) (Wahbeh et al., 2014). The findings underscore the potential of online mindfulness-based interventions to enhance mental health outcomes, particularly in diminishing stress. Online interventions offer several advantages, including cost-effectiveness, flexibility, convenience, and comfort (Spijkerman et al., 2016).

When applied in an individual clinical context, MBCT offers additional benefits such as providing confidentiality for client sessions, more flexible scheduling options, and the ability to adapt sessions more closely to the client’s needs. Research by Schroevers et al. (2016) demonstrated substantial reductions in symptoms of depression and anxiety among participants undergoing both group and individual MBCT, with no significant disparities observed between the two modalities. This evidence supports the effectiveness of MBCT in improving mental health outcomes regardless of the format in which it is delivered.

Our research aims to assess the feasibility of implementing an online mindfulness-based cognitive therapy (eMBCT) program specifically designed for TRD in an individual clinical setting. By exploring this, we hope to contribute to the growing body of evidence supporting the efficacy of digital mental health interventions and expand the therapeutic options available for individuals with TRD, making the most of the benefits of the online format to enhance accessibility and impact.

## 2 Materials and methods

### 2.1 Study design parallel two-arm pilot trial

This research is a two-arm parallel randomized controlled feasibility trial: an 8-week experimental (eMBCT) and a control group (CG). Participant’s enrollment started in April 2020 ending in May 2021. At baseline, self-report measures and written informed consent were obtained from all participants before participating in the study, which was approved by the Research Ethics Committees (The Brazilian Clinical Trials Registry/Registro Brasileiro de Ensaios Clínicos: RBR-6zndpbv); and then were randomized.

This study utilized a flexible approach to online interventions, allowing participants to choose from three different communication platforms for the sessions: Zoom, Google Meet, and WhatsApp Web. This choice was offered to accommodate participant preferences and ensure greater accessibility and comfort during the intervention process. The use of multiple platforms was intended to reflect a real-world setting and enhance participant engagement.

Assessments were completed at the end of intervention where participants in the control group had the chance to receive the same intervention following completion of assessment period. Changes in pharmacological treatment were monitored by psychiatry during the study period, with the possibility of dosage adjustment when necessary. Since this was a pilot study, a sample size calculation was not performed. Random allocation was computer generated. Participants were randomly assigned (1:1) in block sizes of four by computer-generated randomization to the eMBCT group or the control group by a computer-generated randomization.

The control group was placed on a waiting list and received usual care during the study period. Participants in the control group continued to receive usual care, which included any ongoing pharmacological treatments and regular psychiatric consultations. This approach was chosen to control for potential confounding factors and to ensure that any observed effects in the experimental group could be attributed to the eMBCT intervention rather than other variables.

The control group was monitored to ensure adherence to their usual care regimen, and any changes in their pharmacological treatment were documented. This monitoring was crucial to maintain the integrity of the study and to provide a clear comparison between the intervention and control groups. To further ensure the validity of the study, the same self-report measures used in the intervention group were also administered to the control group at baseline and at the end of the intervention period. This allowed for a direct comparison of outcomes between the two groups. The use of a waiting list control group is a common practice in clinical trials and has been shown to be effective in controlling for placebo effects and other confounding variables (Mohr et al., 2009).

### 2.2 Participants

**Figure 1:**
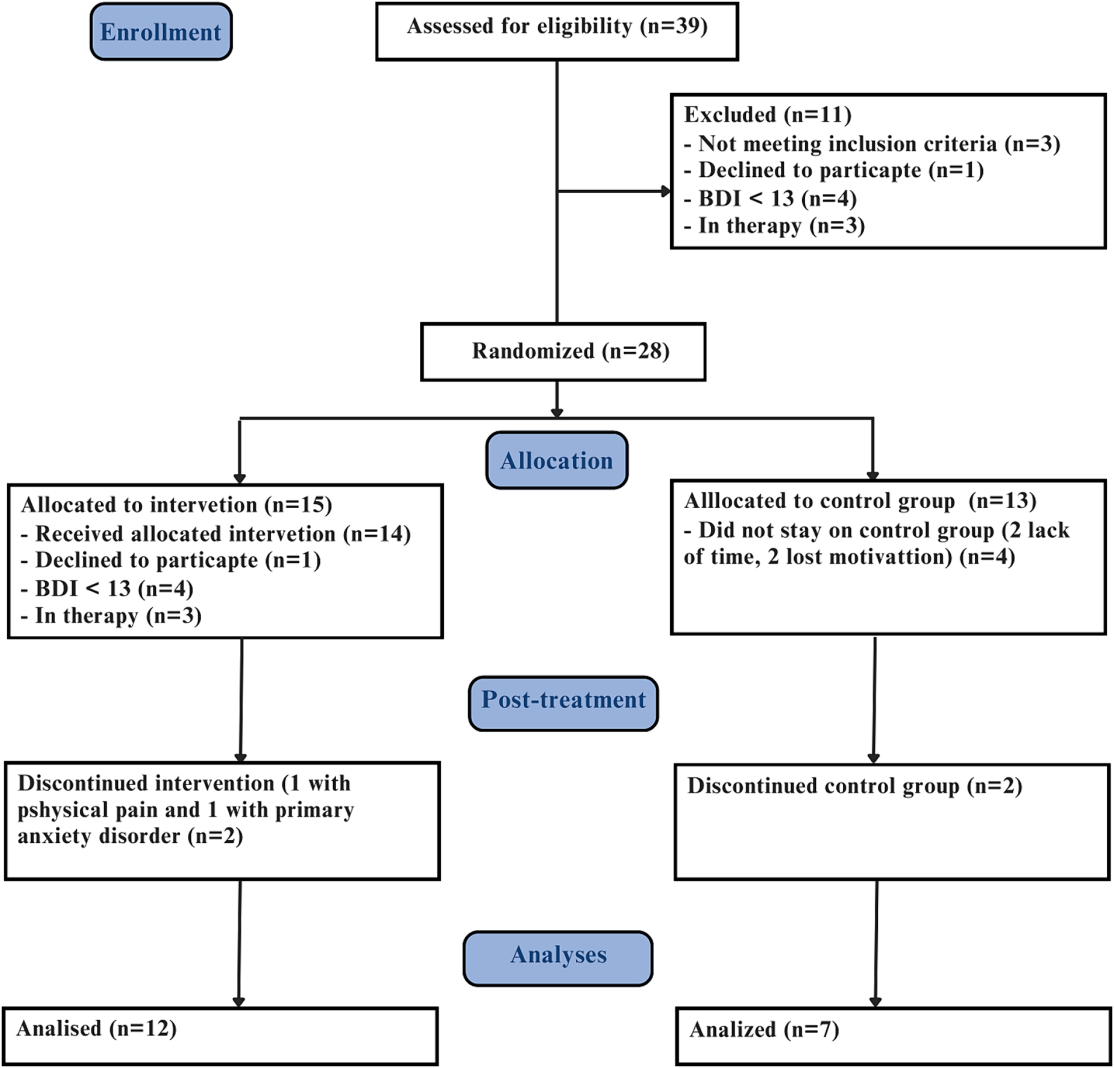
Consort flow diagram.

18 years old subjects or older, identified with TRD from the Outpatient Resistant Depression Unit - Institute of Psychiatry - Federal University of Rio de Janeiro. Inclusion criteria: Moderate to high level of current depressive symptoms (Beck Depression Inventory, BDI ≥ 20), access to a smartphone, laptop, tablet, or computer. Exclusion criteria: were cognitive or mindfulness training within the past year, as well as, current psychotic symptoms, bipolar disorder (Mini International Neuropsychiatric Interview - MINI 5.0.0/DSM IV) (Amorim, 2000). Diagnosis of TRD was performed in person and confirmed by the Massachusetts General Hospital – Antidepressant Treatment Response Questionnaire (MGH-ATRQ). Clinical assessments were obtained by trained psychiatrists and psychologists from the same center (Figure 1).

### 2.3 Intervention

The eMBCT in an individual format was adapted from the traditional MBCT-group with the following modifications: we reduced durations of session to 1 h (original 2.5 h), mindfulness practices during the week were shortened (about 40 min per day) but number of sessions (8 weeks) maintained; psychoeducational content was adapted to participants with TRD. Audio files and mindfulness diaries were sent in downloadable format at the end of each session. The original MBCT program for people at high risk for depressive relapse are described in detail as a treatment manual (Segal et al., 2013).

### 2.4 Instruments

Patients completed a series of validated Portuguese questionnaires before and after the intervention to assess various psychological dimensions. Detailed descriptions of these instruments are provided below:

#### 2.4.1 Beck depression inventory (BDI)

The BDI is a 21-item self-report inventory measuring the severity of depression. Each item is rated on a 4-point scale ranging from 0 to 3, with higher total scores indicating more severe depressive symptoms. The BDI is widely used for its psychometric robustness, including high reliability and validity in clinical and non-clinical populations (Beck et al., 1996).

#### 2.4.2 Beck anxiety inventory (BAI)

This inventory consists of 21 items, each describing a common symptom of anxiety. Respondents rate how much they have been bothered by each symptom during the past week on a scale of 0 (not at all) to 3 (severely). The BAI is known for its excellent psychometric properties, including high internal consistency and good discriminant validity (Beck et al.,1988).

#### 2.4.3 Mindful awareness and attention scale (MAAS)

The MAAS is a 15-item scale designed to assess the frequency of mindful states in day-to-day life. Items are rated on a 6-point Likert scale from 1 (almost always) to 6 (almost never). This instrument has been validated for use in various populations and is valued for its ability to predict well-being and stress-related outcomes (De Barros et al., 2015).

#### 2.4.4 Self-compassion scale (SCS)

Comprising 26 items, the SCS measures the components of self-compassion (self-kindness, common humanity, and mindfulness) versus their counterparts (self-judgment, isolation, and over-identification). Items are rated on a 5-point scale from 1 (almost never) to 5 (almost always). The scale demonstrates excellent reliability and validity, correlating positively with psychological health (Neff, 2003).

#### 2.4.5 Daily diary

Developed by Cunha (2001), this diary method involves daily entries by participants to record their experiences, thoughts, and feelings. Although not a standardized psychometric instrument, it provides qualitative data that enrich the quantitative findings, offering insights into the daily lives and changes in the psychological states of participants.

### 2.5 Statistical analysis

All statistical analyzes were performed using SPSS® software, version 20.0 (IBM Corporation, NY, USA). Sociodemographic variables were described by frequencies and percentages or means and standard deviation, depending on the nature of the data. To compare categorical and continuous variables between subgroups,, the *χ*2 test, Fisher’s exact test or the Mann–Whitney U test were used.

## 3 Results

Thirty-nine participants diagnosed with TRD were assessed for eligibility, resulting in 11 exclusions, in which 3 did not meet the clinical trial inclusion criteria, 4 had BDI score lower than 13, 1 declined participation and 3 were already in therapy. Consequently, 28 participants were randomized in two groups: 15 were allocated to eMBCT, and 13 to the control group (CG). Out of these, 19 participants completed the trial, (12 eMBCT - 7 CG), predominantly female, aged between 24 and 67 years (median 50, IQR 19,5).

The subgroups were found to be homogeneous across sex [(X2 (1) = 1.421; *p* = 0.532], marital status [(X2 (1) = 0.142; *p* = 0.914], work [(X2 (1) =0.819; *p* = 0.415], income [(X2 (3) = 1.954; *p* = 0.711], age (*U* = 48.00; *p* = 0.136) and education level (*U* = 71.00; *p* = 0.954).

**Figure 2:**
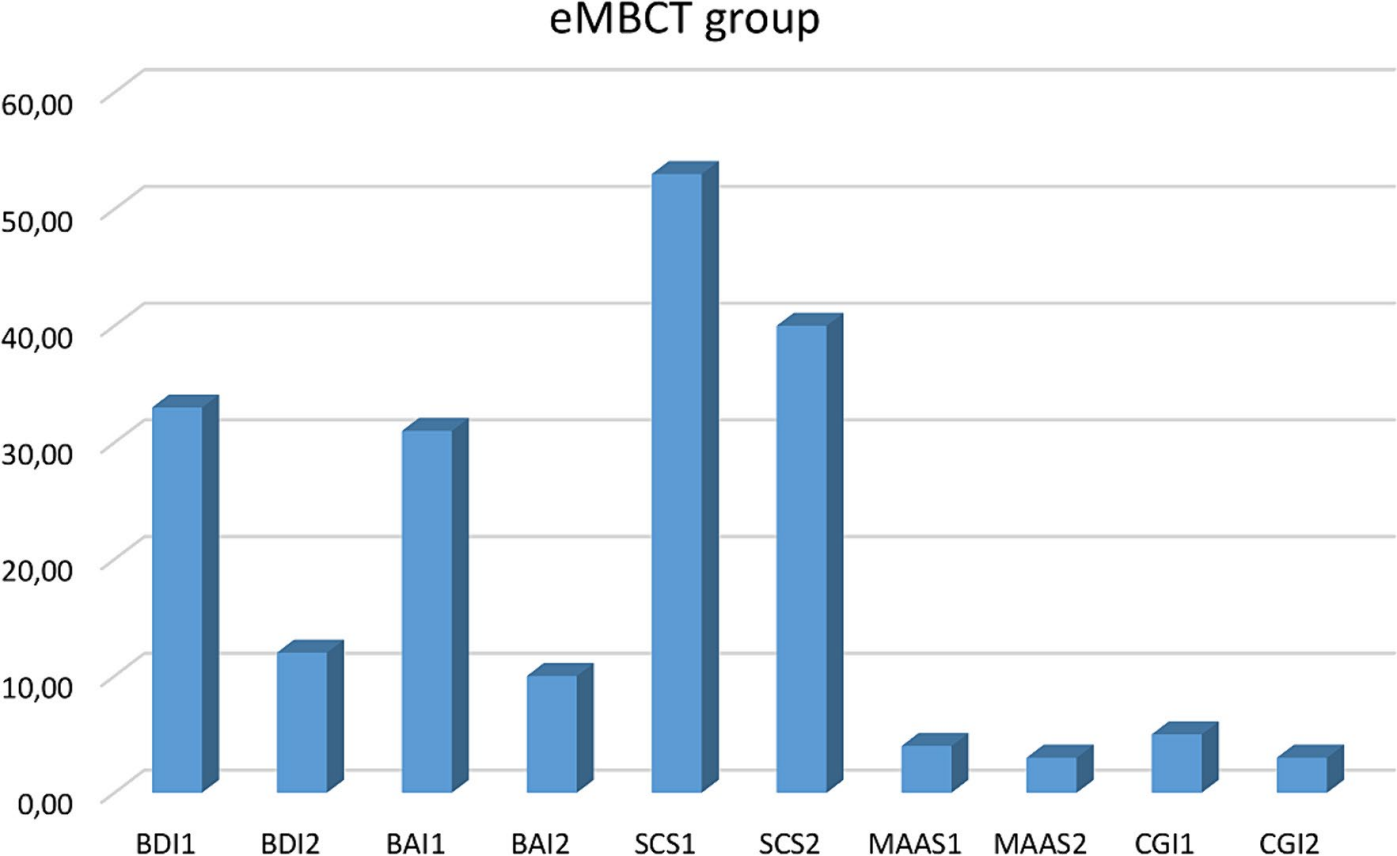
Clinical variables comparison before and after the mindfulness intervention. BDI, Beck Depression Inventory; BAI, Beck Anxiety Inventory; SCS, Self-Compassion Scale; MAAS, Mindful Attention Awareness Scale. CGI, Clinical Global Impression.

**Figure 3:**
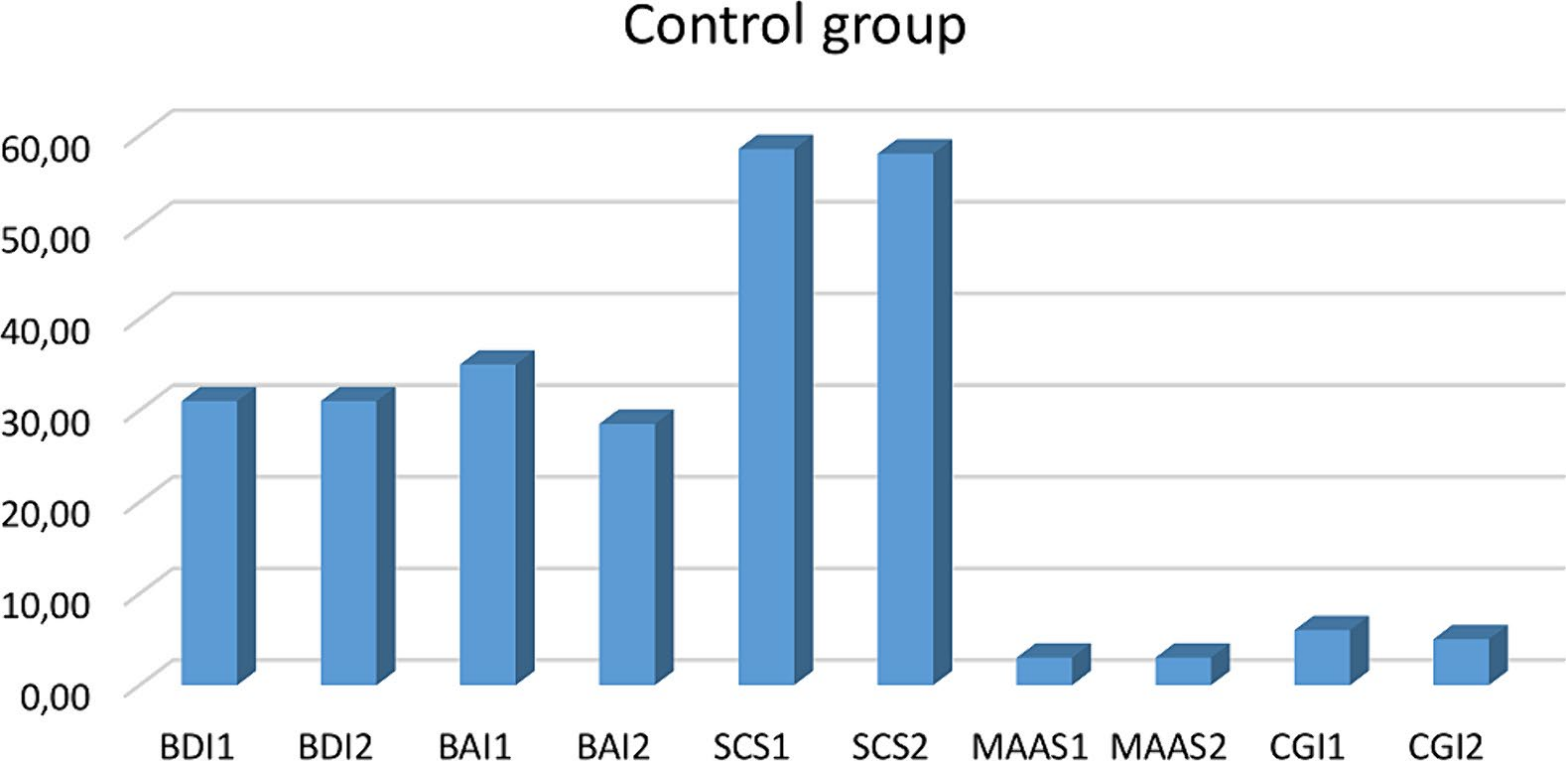
Clinical variable comparison in the control group. BDI, Beck Depression Inventory; BAI, Beck Anxiety Inventory; SCS, Self-Compassion Scale; MAAS, Mindful Attention Awareness Scale. CGI, Clinical Global Impression.

**Figure 4:**
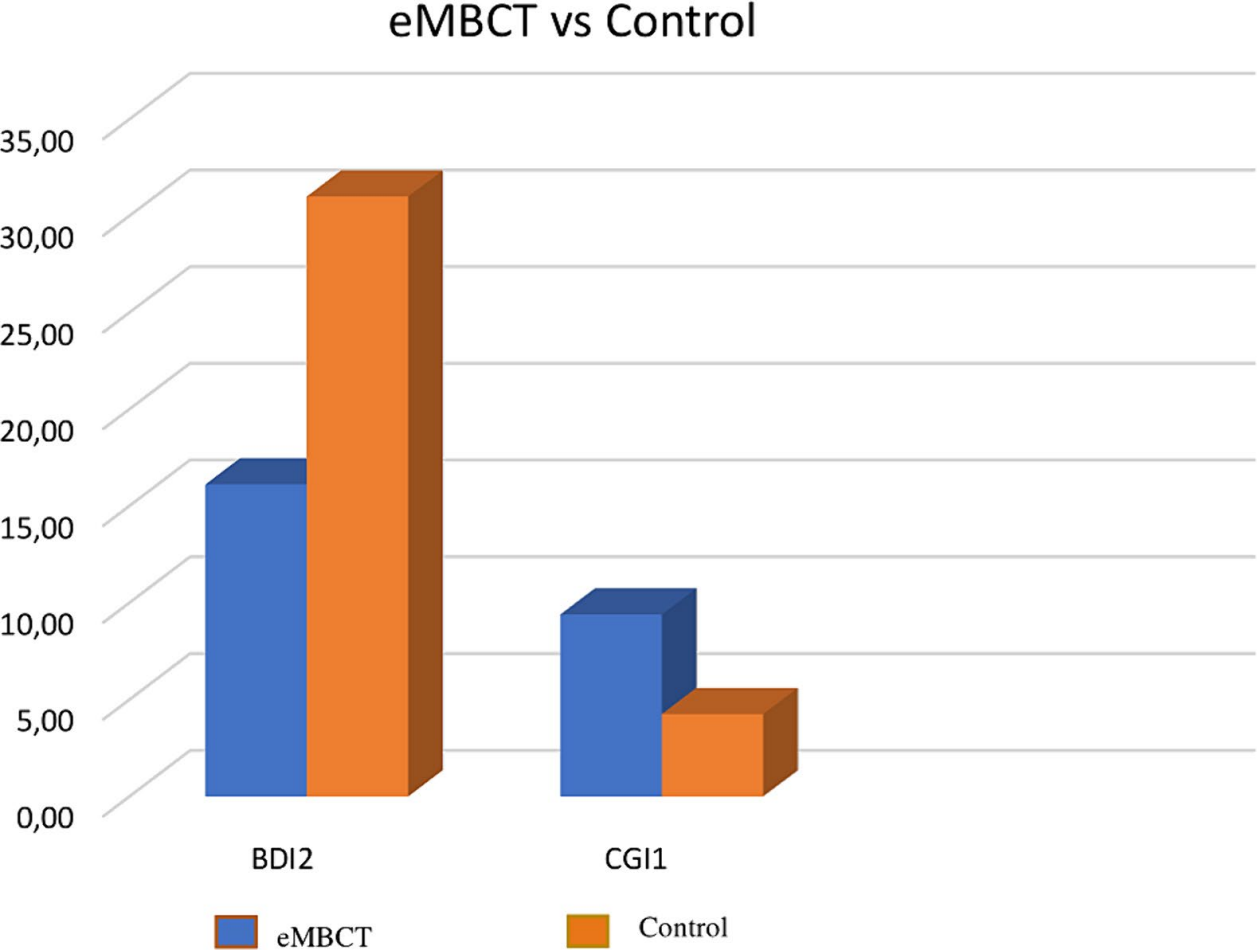
Comparison of eMBCT Group and Control Group on BDI and CGI Scores.

Intragroup, the Wilcoxon test showed eMBCT group differences: BDI2 - BDI1 (*Z* = −3.423; *p* = 0.001); effect size *r* = 0.78, BAI2-BAI1 (*Z* = −3.361; *p* = 0.001); effect size *r* = 0.77, MAAS2 - MAAS1(*Z* = − 3.125; *p* = 0.002); effect size *r* = 0.32, CGI2 - CGI1(*Z* = −2.546; *p* = 0.011); effect size *r* = 0.25). No significant changes were observed in any variable within the control group (Tables 1, 2; Figures 1–4).

**Table 1:** Mean and standard deviation of related samples (pre-test and post-test) in the eMBCT.

	*n*	Pre	Post	*Z*	*p*	*r*
BDI	19	33.05 (11.1)	16.1 (11.9)	−3.423	0.001	0.78
BAI	19	33.4 (15.1)	16.2 (12.9)	−3.361	0.001	0.77
CGI	10	9.0 (5.8)	3.0 (1.0)	−2.546	0.011	0.25
MAAS	19	6.1 (4.1)	2.9 (1.3)	−3.125	0.002	0.32
SCS	19	52.6 (14.0)	40.0 (31.6)	−2.246	0.122	–

BDI = Beck Depression Inventory; BAI = Beck Anxiety Inventory; CGI = Clinical Global Impression; MAAS = Mindful Attention Awareness Scale; SCS = Self-Compassion Scale.

**Table 2:** Mean and standard deviation of related samples (pre-test and post-test) in the control group.

	*n*	Pre	Post	*Z*	*p*	*r*
BDI	8	31.8 (5.9)	31.0 (11.5)	−0.135	0.001	–
BAI	8	33.6 (13.4)	28.1 (16.2)	−0.561	0.001	–
CGI	6	6.3 (3.4)	5.5 (2.3)	−1.511	0.011	–
MAAS	8	3.7 (2.6)	3.2 (1.6)	−1.000	0.002	–
SCS	8	57.6 (16.0)	55.3 (21.6)	−0.350	0.122	–

BDI = Beck Depression Inventory; BAI = Beck Anxiety Inventory; CGI = Clinical Global Impression; MAAS = Mindful Attention Awareness Scale; SCS = Self-Compassion Scale.

Intergroup comparisons using the non-parametric Mann–Whitney U identified a statistical differences between the eMBCT and control groups for BDI2 (*U* = 30.5; *p* = 0.015; effect size *r* = 0.47) and CGI1 (*U* = 21.0; *p* = 0.004; effect size *r* = 0.56). No statistical differences were observed for the other variables: BDI1 (*U* = 75.5; *p* = 0.979), BAI1 (*U* = 73.5; *p* = 0.894), BAI2 (*U* = 44.5; *p* = 0.069), SCS1 (*U* = 63.5; *p* = 0.506), SCS2 (*U* = 60.5; *p* = 0.410), MAAS1 (*U* = 42.0; *p* = 0.065), MAAS2 (*U* = 69.5; *p* = 0.886), SheF2 (*U* = 18.0; *p* = 0.038), and SheS2 (*U* = 31.0; *p* = 0.020).

Time Interaction analysis through a two-way mixed ANOVA demonstrated that there is an effect of time in the intragroup comparison for the variables BDI [*F*(1, 24 = 9.226; p< 0.05)], BAI [*F*(1, 24 = 9.226; p< 0. 05)], MAAS [*F*(1, 24 = 6.539; p< 0.005)]. Meanwhile, the intergroup comparison demonstrated that there is an effect of the interventions on the BDI variable [*F*(1, 24 = 676.08; p< 0.05)].

Detailed reporting of attrition rates and adverse events is crucial for assessing the reliability and safety of the study. In the intervention group, one participant did not complete the trial due to lack of time. In the control group, two participants did not participate due to lack of time, and two others lost motivation. Importantly, no participants reported issues related to the online nature of the intervention, such as discomfort with online treatment or technical issues. This suggests that the digital format of eMBCT was well-received and did not pose significant barriers to participation.

No adverse events were reported during the study, indicating that the eMBCT intervention was safe for the participants. The absence of technical issues or discomfort related to the online format further supports the feasibility of delivering MBCT digitally.

## 4 Discussion

This randomized controlled trial (RCT) aims to add to the growing body of knowledge in the digital mental health domain by assessing the effectiveness of an e-MBCT program tailored for individuals with TRD. Our findings indicate a statistically significant decrease in depressive symptoms among the eMBCT group participants, with 26% experiencing complete remission. These results suggest that eMBCT, when provided in a digital format, could offer a viable option for those who have found limited success with standard treatments.

Our research aligns with and extends the findings of previous studies, such as Schroevers et al. (2016), who found MBCT to be effective in reducing depression, and supports the perspective of Spijkerman et al. (2016) regarding the significance of digital interventions in mental health care. Additionally, our observations are consistent with those of Eisendrath et al. (2011), and Cladder-Micus et al. (2018), who noted improvements in depressive symptoms among TRD patients following MBCT, further affirming the value of mindfulness-based approaches.

From a psychological and clinical perspective, our research highlights the integration between mindfulness practices and aspects of cognitive-behavioral therapy for depression (Beck et al.,1979). This combination is essential for disengaging individuals from the repetitive nature of automatic negative thoughts characteristic of depression (MacKenzie et al., 2018). The adaptability of MBCT to an online format enhances its accessibility and applicability, suggesting a promising avenue for future clinical use and research within the field of mental health treatment.

Our findings are further supported by studies demonstrating the effectiveness of MBCT in various settings. For instance, Kuyken et al. (2016) showed the efficacy of face-to-face MBCT in preventing depressive relapse, while Piet and Hougaard (2011) conducted a meta-analysis confirming the benefits of group-based MBCT for recurrent depression. These studies collectively illustrate that MBCT’s effectiveness is not confined to a single delivery method but extends across different formats, including digital platforms.

The significant improvement within the eMBCT group, as opposed to the control group, can be explained by several specific mechanisms inherent to the eMBCT intervention. eMBCT emphasizes mindfulness practices that help individuals become more aware of their thoughts and feelings in a non-judgmental way, allowing them to recognize and disengage from automatic negative thoughts (Kabat-Zinn, 1990). Additionally, eMBCT fosters self-compassion, encouraging individuals to treat themselves with kindness and understanding during difficult times, which reduces self-criticism and promotes emotional resilience (Neff, 2003). These mechanisms are crucial for managing depressive symptoms and were not present in the control group, which did not receive similar training. Furthermore, the integration of cognitive-behavioral strategies with mindfulness in eMBCT helps participants reframe negative thought patterns and develop healthier cognitive responses (Segal et al., 2002). The digital format of eMBCT also provides greater accessibility and flexibility, allowing participants to attend sessions without the need for physical travel and offering more scheduling options, which likely contributed to the significant improvements observed within the eMBCT group.

Acknowledging the limitations, the small and predominantly female sample size of our study may limit the generalizability of our findings. The short study duration, which could have been extended to include more groups, thereby achieving a larger sample size, is another significant limitation. Variations in medication during the trial period and the absence of a structured, manualized control intervention might have impacted the outcomes. Additionally, the variability from employing different psychologists, despite uniform training, are notable limitations. The non-significant findings in mindfulness and self-compassion scales highlight the need for more comprehensive future research.

These factors may have influenced the results and should be addressed in future research to enhance the robustness and applicability of the findings. Future studies should consider a longer duration, a larger and more diverse sample size, and the implementation of a standardized control intervention to mitigate these limitations. On the other hand, the study’s strengths lie in its pioneering role in exploring eMBCT for TRD within a RCT structure, marking a significant contribution to the rapidly growing evidence base supporting digital mental health interventions. The consistency in training among psychologists delivering the intervention reduces variability, promoting a standardization approach to treatment (Segal et al., 2013).

Our results offer useful insights for both research and clinical practice, suggesting the potential of digital MBCT applications in treating TRD, proposing for the integration of eMBCT into broader treatment modalities. This approach aligns with the alternative toward telehealth and digital mental health services, offering a viable, cost-effective, and accessible option that could work alongside (Spijkerman et al., 2016).

Future research should focus on consolidating the online individual format of eMBCT. This could include the development of supplementary tools, such as mobile applications, to enhance engagement and provide additional support between sessions. Exploring the integration of more frequent or extended sessions could also be beneficial in maximizing the therapeutic impact. Additionally, investigating the long-term effects of eMBCT is crucial to understand its sustainability and potential for preventing relapse. Longitudinal studies with follow-up assessments at multiple intervals post-intervention would provide valuable insights into the enduring benefits of eMBCT.

Moreover, qualitative research could provide deeper insights into how patients experience this form of therapy, potentially leading to improvements in how digital MBCT is utilized for treating TRD and related conditions. Understanding patient perspectives can help tailor the program to better meet their needs and enhance engagement.

Implementing eMBCT in actual clinical practice presents several challenges and opportunities. One significant advantage is the elimination of geographical barriers, making it accessible to individuals in remote areas. However, ensuring consistent internet access and digital literacy among participants is essential for effective implementation. Training clinicians to deliver eMBCT with fidelity is another critical factor, as variability in delivery can impact outcomes. Developing standardized training programs and certification processes for eMBCT practitioners can help maintain the quality and consistency of the intervention.

In conclusion, this study suggests the effectiveness of an online MBCT program in an individual format as an adjunctive treatment for TRD, indicating a positive step forward in expanding therapeutic choices. Future research should focus on studies to engage with larger and more varied participant groups of RCT research to better understand the enduring effects of eMBCT. Further qualitative research could provide deeper insights into how patients experience this form of therapy, potentially leading to improvements in how digital MBCT is utilized for treating TRD and related conditions. The ongoing investigation and improvement of digital mental interventions such as eMBCT hold promise for enhancing the strategies available for managing TRD, highlighting the value of continued research in this field.

## Data availability statement

The original contributions presented in the study are included in the article/supplementary material, further inquiries can be directed to the corresponding author.

## Ethics statement

The studies involving humans were approved by the Brazilian Clinical Trials Registry: RBR-6zndpbv. The studies were conducted in accordance with the local legislation and institutional requirements. The participants provided their written informed consent to participate in this study.

## Author contributions

MR: Writing – original draft, Methodology, Investigation, Conceptualization. LQ: Writing – original draft, Data curation. JA: Writing – review & editing, Supervision. AN: Writing – review & editing, Supervision, Project administration.

## References

[ref1] Al-harbi K. S. (2012). Treatment-resistant depression: therapeutic trends, challenges, and future directions. Patient Prefer. Adherence 6, 369–388. doi: 10.2147/ppa.s29716, PMID: 22654508 PMC3363299

[ref2] Amorim P. (2000). Mini International Neuropsychiatric Interview (MINI): validação de entrevista breve para diagnóstico de transtornos mentais. Rev. Bras. Psiquiatr. 22, 106–115. doi: 10.1590/s1516-44462000000300003

[ref700] Beck A. T. Rush A. J. Shaw B. F. Emery G. (1979). Cognitive Therapy of Depression. New York: Guildford Press.

[ref9001] Beck A. T. Steer R. A. Brown G. K., (1996). Manual for the Beck Depression Inventory-II. San Antonio, TX: Psychological Corporation.

[ref9002] Beck A. T. Steer R. A. Garbin M. G. (1988). Psychometric properties of the Beck Depression Inventory: Twenty-five years of evaluation. Clin. Psychol. Rev, 8, 77–100.

[ref3] Cladder-Micus M. B. Speckens A. E. M. Vrijsen J. N. Hertel P. Becker E. S. Spinhoven P. (2018). Mindfulness-based cognitive therapy for patients with chronic, treatment-resistant depression: a pragmatic randomized controlled trial. Dep. anxiety 35, 914–924. doi: 10.1002/da.22788, PMID: 30088834 PMC6175087

[ref4] Cuijpers P. Berking M. Andersson G. Quigley L. Kleiboer A. Dobson K. S. (2013). A meta-analysis of cognitive-behavioural therapy for adult depression, alone and in comparison with other treatments. Can. J. Psychiatry 58, 376–385. doi: 10.1177/070674371305800702, PMID: 23870719

[ref5] Cuijpers P. Vogelzangs N. Twisk J. Kleiboer A. Li J. Penninx B. W. (2014). Comprehensive meta-analysis of excess mortality in depression in the general community versus patients with specific illnesses. Am. J. Psychiatry 171, 453–462. doi: 10.1176/appi.ajp.2013.13030325, PMID: 24434956

[ref6] Cunha J. A. (2001). Manual da versão em português das Escalas Beck. São Paulo: Casa do Psicólogo.

[ref7] De Barros V. V. Kozasa E. H. Souza I. C. W. Ronzani T. M. (2015). Validity evidence of the brazilian version of the mindful attention awareness scale (MAAS). Psicologia Reflexão e Crítica 28, 87–95. doi: 10.1590/1678-7153.201528110

[ref8] Eisendrath S. Chartier M. McLane M. (2011). Adapting mindfulness-based cognitive therapy for treatment-resistant depression. Cogn. Behav. Pract. 18, 362–370. doi: 10.1016/j.cbpra.2010.05.004, PMID: 22211062 PMC3247069

[ref9] Gaynes B. N. Lux L. J. Lloyd S. W. Hansen R. A. Gartlehner G. Keener P.. (2011). Nonpharmacologic interventions for treatment-resistant depression in adults. Rockville (MD): Agency for Healthcare Research and Quality (US). 22091472

[ref9004] Kabat-Zinn J. (1990). Full Catastrophe Living: Using the Wisdom of Your Body and Mind to Face Stress, Pain, and Illness. New York: Delacorte.

[ref10] Kuyken W. Warren F. C. Taylor R. S. Whalley B. Crane C. Bondolfi G.. (2016). Efficacy of mindfulness-based cognitive therapy in prevention of depressive relapse: an individual patient data meta-analysis from randomized trials. JAMA Psychiatry 73, 565–574. doi: 10.1001/jamapsychiatry.2016.0076, PMID: 27119968 PMC6640038

[ref11] Lundberg J. Cars T. Lampa E. Ekholm Selling K. Leval A. Gannedahl A.. (2023). Determinants and outcomes of suicidal behavior among patients with major depressive disorder. JAMA Psychiatry 80, 1218–1225. doi: 10.1001/jamapsychiatry.2023.2833, PMID: 37585196 PMC10433143

[ref12] MacKenzie M. Abbott K. Kocovski N. (2018). Mindfulness-based cognitive therapy in patients with depression: current perspectives. Neuropsychiatr. Dis. Treat. 4, 1599–1605. doi: 10.2147/ndt.s160761, PMID: 29950842 PMC6018485

[ref13] McIntyre R. S. Alsuwaidan M. Baune B. T. Berk M. Demyttenaere K. Goldberg J. F.. (2023). Treatment-resistant depression: definition, prevalence, detection, management, and investigational interventions. World psychiatry 22, 394–412. doi: 10.1002/wps.21120, PMID: 37713549 PMC10503923

[ref14] Mohr D. C. Spring B. Freedland K. E. Beckner V. Arean P. Hollon S. D.. (2009). The selection and Design of Control Conditions for randomized controlled trials of psychological interventions. Psychother. Psychosom. 78, 275–284. doi: 10.1159/000228248 19602916

[ref15] Monnart A. Vanderhasselt M. A. Schroder E. Campanella S. Fontaine P. Kornreich C. (2019). Treatment of resistant depression: a pilot study assessing the efficacy of a tDCS-mindfulness program compared with a tDCS-relaxation program. Front. psychiatry 10:730. doi: 10.3389/fpsyt.2019.00730, PMID: 31708808 PMC6819945

[ref9003] Neff K. D. (2003). The development and validation of a scale to measure self-compassion. Self and Identity, 2, 223–250.

[ref16] Piet J. Hougaard E. (2011). The effect of mindfulness-based cognitive therapy for prevention of relapse in recurrent major depressive disorder: a systematic review and meta-analysis. Clinic. Psychol. Rev. 31, 1032–1040. doi: 10.1016/j.cpr.2011.05.002, PMID: 21802618

[ref17] Ramos G. Hernandez-Ramos R. Taylor M. Schueller S. M. (2024). State of the science: using digital mental health interventions to extend the impact of psychological services. Behav. Ther. doi: 10.1016/j.beth.2024.04.004 39443071

[ref18] Rush A. J. Trivedi M. H. Wisniewski S. R. Nierenberg A. A. Stewart J. W. Warden D.. (2006). Acute and longer-term outcomes in depressed outpatients requiring one or several treatment steps: a STAR*D report. Americ. J. Psychiatry 163, 1905–1917. doi: 10.1176/ajp.2006.163.11.1905 17074942

[ref19] Salloum N. C. Papakostas G. I. (2019). Staging treatment intensity and defining resistant depression: historical overview and future directions. J. Clin. Psychiatry 80:21624. doi: 10.4088/JCP.18r12250, PMID: 31163105

[ref20] Schroevers M. J. Tovote K. A. Snippe E. Fleer J. (2016). Group and individual mindfulness-based cognitive therapy (MBCT) are both effective: a pilot randomized controlled trial in depressed people with a somatic disease. Mindfulness 7, 1339–1346. doi: 10.1007/s12671-016-0575-z, PMID: 27909465 PMC5107193

[ref21] Segal Z. V. Williams J. M. Teasdale J. D. (2013). Mindfulness-based cognitive therapy for depression: A new approach to preventing relapse.New. York, NY: Guilford Press.

[ref22] Segal Z. V. Williams J. M. G. Teasdale J. D. (2002). Mindfulness-based cognitive therapy for depression: A new approach to preventing relapse. New York, NY: The Guilford Press.

[ref23] Spijkerman M. P. J. Pots W. T. M. Bohlmeijer E. T. (2016). Effectiveness of online mindfulness-based interventions in improving mental health: a review and meta-analysis of randomised controlled trials. Clin. Psychol. Rev. 45, 102–114. doi: 10.1016/j.cpr.2016.03.009, PMID: 27111302

[ref24] Voineskos D. Daskalakis Z. J. Blumberger D. M. (2020). Management of Treatment-Resistant Depression: challenges and strategies. Neuropsychiatr. Dis. Treat. 16, 221–234. doi: 10.2147/ndt.s198774, PMID: 32021216 PMC6982454

[ref25] Wahbeh H. Svalina M. N. Oken B. S. (2014). Group, one-on-one, or internet? Preferences for mindfulness meditation delivery format and their predictors. Open Med. J. 1, 66–74. doi: 10.2174/1874220301401010066, PMID: 27057260 PMC4820831

